# Deciphering Acute Coronary Syndromes Pathobiology Through Proteomics

**DOI:** 10.3390/jcdd12050188

**Published:** 2025-05-15

**Authors:** Gabriele Nieddu, Marilena Formato, Antonio Junior Lepedda

**Affiliations:** Department of Biomedical Sciences, University of Sassari, 07100 Sassari, Italy; ganieddu@uniss.it (G.N.); formato@uniss.it (M.F.)

**Keywords:** CVD, proteomics, ACS biomarkers, plaque vulnerability

## Abstract

Acute coronary syndrome (ACS) refers to a spectrum of conditions characterized by a sudden decrease in blood flow to the heart. This includes unstable angina, the mildest form, as well as non-ST- and ST-segment elevation myocardial infarction. The primary cause of ACS is typically the rupture or erosion of an atherosclerotic plaque in a coronary artery, resulting in the formation of a blood clot that can, partially or completely, block the blood flow to the heart muscle. The ongoing discovery and comprehension of emerging biomarkers for atherosclerosis could enhance our capacity to predict future events, particularly when integrated alongside traditional risk factors in assessing overall risk profiles. With advancements in proteomic technologies, large-scale approaches have been increasingly instrumental in unraveling pathways implicated in atherosclerotic degeneration and identifying novel circulating markers, which may serve as early diagnostic indicators or targets for innovative therapies. Over recent decades, numerous matrices including plasma, urine, microparticles, lipoproteins, atherosclerotic plaque extracts and secretomes, as well as thrombi, have been examined to address these questions. Furthermore, proteomics has been applied to various experimental models of atherosclerosis to deepen our understanding of the mechanisms underlying atherogenesis. This review offers a critical overview of the past two decades of untargeted omics research focused on identifying circulating and tissue biomarkers relevant to ACS.

## 1. Introduction

**Figure 1:**
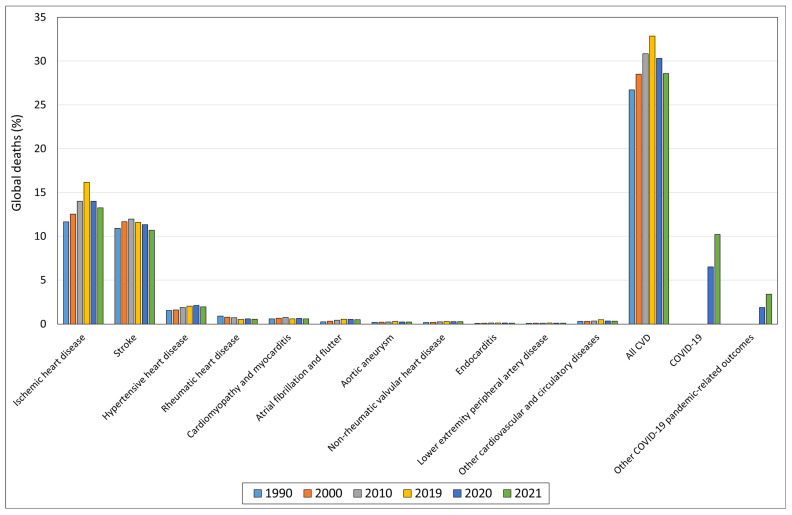
Trends of CVD-related deaths over the past three decades and impact of COVID-19 and COVID-19-related deaths in the pandemic biennium. “https://vizhub.healthdata.org/gbd-compare/” (accessed on 28 January 2025).

Despite significant advancements in diagnosis and treatment, cardiovascular disease (CVD) remains the leading cause of death and illness globally, with a higher prevalence observed in developed countries [1]. In 2019, CVD accounted for approximately 33% of global deaths, a figure that slightly decreased to 29% in 2021, representing ischemic heart disease and stroke as the main contributors. Particularly concerning is the upward trend observed over the last three decades (1990–2019), with only an apparent decline during the COVID-19 pandemic years, likely influenced in part by COVID-19 and COVID-19-related fatalities (Figure 1).

**Figure 2:**
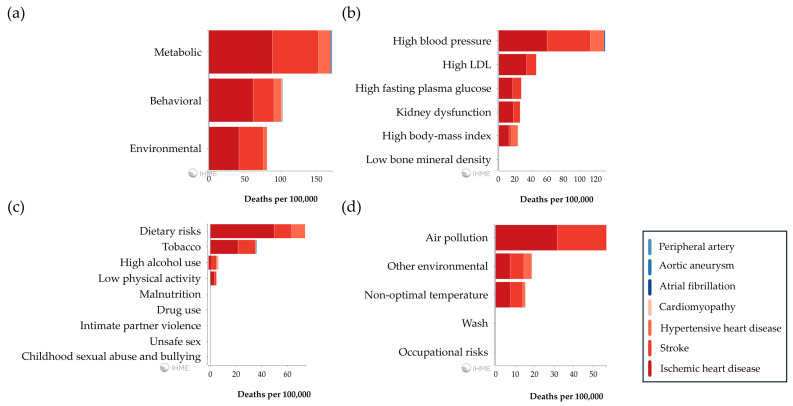
Contribution of known risk factors to CVD-related global deaths in 2021 (**a**) sorted into metabolic (**b**), behavioral (**c**), and environmental (**d**). “https://vizhub.healthdata.org/gbd-compare/” (accessed on 28 January 2025).

Several metabolic, behavioral, and environmental risk factors are known to contribute to overall CVD-related global deaths (Figure 2).

Currently, cardiovascular risk prediction, which still relies on traditional risk factors, can be unsatisfactory. In this scenario, identifying circulating biomarkers of subclinical atherosclerosis is of critical importance to improve cardiovascular risk prediction and early intervention with targeted therapies, potentially reversing/slowing disease progression as well as preventing major adverse cardiovascular events.

Acute coronary syndrome (ACS) refers to a spectrum of conditions characterized by a sudden decrease in blood flow to the heart. It includes unstable angina, the mildest form, as well as non-ST-segment elevation myocardial infarction (NSTEMI) and ST-segment elevation myocardial infarction (STEMI) [2]. ACS is primarily caused by the rupture or erosion of an atherosclerotic plaque within a coronary artery, resulting in the formation of a blood clot that can partially or completely block blood flow to the heart muscle. The presence of coronary plaques may cause chest pain or discomfort, known as angina, which can be classified as stable or unstable depending on its severity. Additional symptoms may include shortness of breath, sweating, nausea, lightheadedness, or fainting. Early recognition and timely treatment are critical to limit heart muscle damage, reduce complications, and improve patient outcomes.

**Figure 3:**
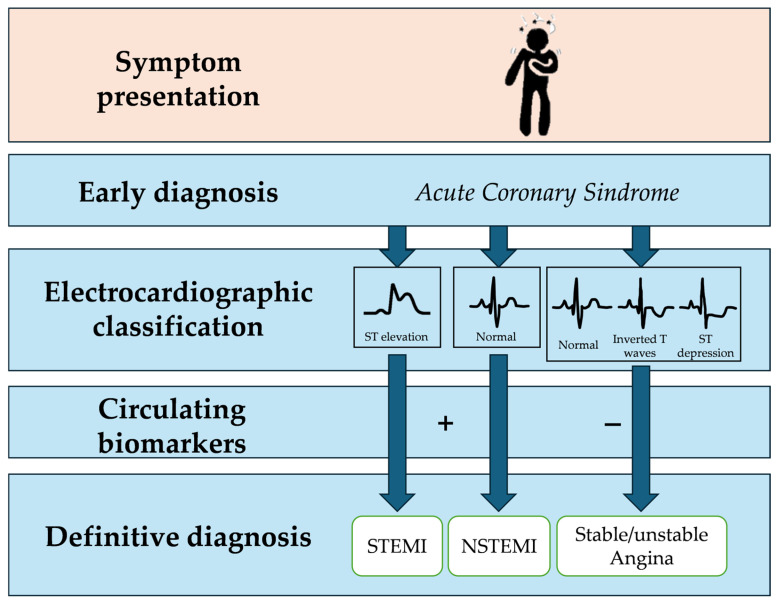
ACS diagnosis through a combination of clinical evaluation, electrocardiography (ECG), and biochemical markers testing.

ACS diagnosis relies on a combination of clinical evaluation, electrocardiography (ECG), and blood tests [3]. However, symptoms such as chest pain are atypical or absent in approximately one-third of patients with ACS, and ST-elevation may also be missing. These factors can contribute to delays in diagnosis and management (Figure 3).

An early diagnosis at admission is crucial for promptly identifying patients with symptoms suggestive of acute CVD. To achieve this, various specific circulating biomarkers, released into the plasma following acute cardiovascular events, are utilized, including brain natriuretic peptide [4,5], high-sensitivity C-reactive protein (hs-CRP) [6,7,8], heart-type fatty acid binding protein (H-FABP) [9,10], creatine kinase-MB (CK-MB) [11,12], and high-sensitivity cardiac troponin (hs-cTn) [13,14,15]. As current tests for diagnosing ACS are not definitive, coronary angiography is often performed, carrying inherent risks associated with this invasive procedure. Consequently, developing non-invasive, multi-marker-based strategies with improved specificity and sensitivity would be both clinically and economically beneficial [16]. Treatment focuses on restoring blood flow to the heart muscle and minimizing myocardial damage. This includes medications such as antiplatelet agents (e.g., aspirin, clopidogrel), anticoagulants (e.g., heparin, enoxaparin), nitroglycerin, beta-blockers, angiotensin-converting enzyme inhibitors, and statins. For patients with STEMI, revascularization procedures, including percutaneous coronary intervention or coronary artery bypass grafting, may be required to reestablish blood flow to the affected heart tissue [17].

Atherosclerosis is a chronic inflammatory condition characterized by the buildup of plaque in the arteries, driven by an interplay between genetic and environmental risk factors such as hypertension, diabetes, hyperlipidemia, high-fat diets, smoking, and physical inactivity [18].

The physiological coronary artery wall comprises three concentric layers: the tunica intima (facing the vessel lumen), the tunica media, and the tunica adventitia. The tunica intima consists of a continuous monolayer of endothelial cells anchored to a basement membrane, playing vital roles in vascular homeostasis, coagulation, inflammation, and tissue growth and repair. Endothelial dysfunction is an early event in atherosclerotic lesion development, marked by the overexpression of adhesion molecules and the secretion of pro-inflammatory and chemotactic cytokines [19,20]. Beneath the intima lies the tunica media, a thick, contractile layer of smooth muscle cells (SMCs) responsible for vessel structural integrity and blood flow and pressure regulation. SMCs contribute to atherogenesis through events such as migration, proliferation, and extracellular matrix (ECM) secretion. During plaque formation, SMCs migrate into the intima, where they proliferate and produce ECM proteins, thickening the arterial wall and narrowing the lumen, which reduces blood flow. Additionally, SMCs can internalize oxidized low-density lipoprotein (LDL), transforming themselves into foam cells that contribute to the formation of the necrotic core. SMCs may also adopt osteoblast-like or chondrocyte-like phenotypes, promoting vascular calcification [21]. The outermost layer, the tunica adventitia, contains a collagen-rich ECM that provides tensile strength to the vessel.

Among the well-established risk factors for atherosclerosis, elevated plasma levels of cholesterol-rich lipoproteins, particularly low-density lipoproteins (LDLs), are closely linked to the early stages of lesion formation [22]. LDL particles can become trapped in the subendothelial space of arteries through specific interactions with ECM components, leading to the formation of fatty streaks [23]. These fatty streaks represent the first visible sign of the atherosclerotic process and can progress toward the formation of plaque, which exhibits considerable variability between individuals in terms of growth rate and pathological characteristics [24]. The erosion or rupture of advanced plaques can trigger thrombosis, resulting in acute clinical events such as ACS, stroke, or peripheral artery occlusion [25,26].

Significant efforts have been dedicated to understanding the factors that contribute to plaque vulnerability and to identifying reliable and specific markers that predict a plaque’s susceptibility to ulceration to prevent adverse clinical events associated with thrombosis and arterial occlusion [27]. Evidence indicates that unstable plaques are characterized by heightened inflammation, increased proteolytic activity [28,29], and a pro-oxidant environment [30,31].

**Figure 4:**
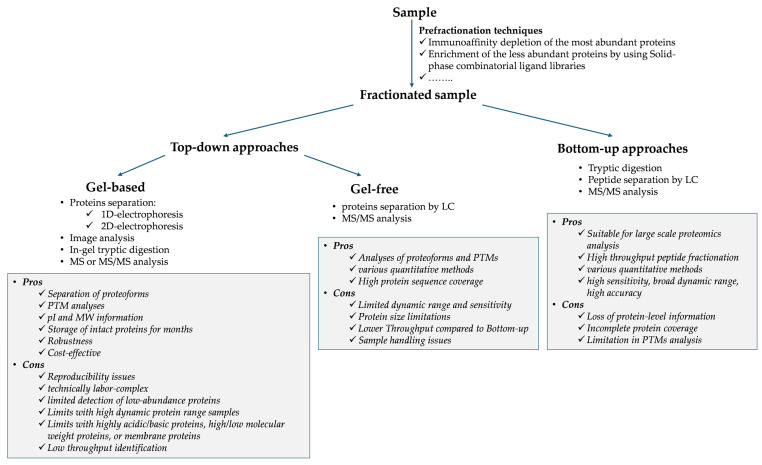
Workflow of untargeted proteomics approaches. Samples can be processed as a whole or after one or more specific prefractionation steps (e.g., immunoaffinity depletion or enrichment procedures are frequently used for plasma samples). In the top-down approach, the whole proteins are separated by one-dimensional (1D) or two-dimensional (2D) polyacrylamide gels (gel-based approaches), or by liquid chromatography (LC) (gel-free approaches), followed by mass spectrometry (MS) or MS/MS analyses. In the bottom-up approach, proteins are fragmented by tryptic digestion and the resulting peptides are separated by LC followed by MS/MS analyses. The main pros and cons of each approach are highlighted.

Over the past two decades, advancements in proteomic technologies have facilitated large-scale investigations into the pathways driving atherosclerotic degeneration. Through the comprehensive analysis of protein expression, modifications, and interactions, proteomics represents a powerful tool for discovering more sensitive and specific biomarkers, contributing to CVD management in clinical practice by enabling earlier diagnosis, personalized risk stratification, and more targeted therapy [32,33,34,35]. To address this challenge, various untargeted proteomics technologies have been employed, including one-dimensional and two-dimensional electrophoresis (1-DE and 2-DE) combined with mass spectrometry (MS) analyses, and gel-free MS-based proteomics (Figure 4). These methods have been applied to a wide range of human-derived matrices [36], such as vascular cells [37,38,39], arterial tissues [40], laser-capture microdissected samples [41], secretomes [42], plasma/serum [43], urine [44], and microparticles [45,46,47]. Platelets, key regulators of inflammation, immune responses [48,49], and targets for thrombotic event prevention, have also been studied using diverse omics approaches, including proteomics, lipidomics, metabolomics, transcriptomics, and translatomics [50]. Proteomics has been further applied to animal models, such as pigs, to deepen our understanding of coronary atherogenesis [51,52]. Emerging lipidomics studies of plasma and lipoproteins [53,54,55], as well as atherosclerotic tissues [56,57], have complemented proteomics, offering additional insights into atherosclerosis progression and plaque degeneration. Additionally, powerful targeted high-throughput proteomics platforms, including Olink and SomaScan, have been gaining traction recently, allowing for thousands of protein measurements simultaneously [58].

This chapter reviews and critically evaluates the major findings of the past 20 years, providing an overview of untargeted proteomics’ contribution to understanding the pathobiology of ACS.

## 2. Identification of ACS Circulating Biomarkers Through Untargeted MS-Based Technologies

Plasma is one of the most valuable matrices for discovering new biomarkers, as it contains both resident proteins, which make up the majority, and proteins released from vascular cells and tissues. However, plasma proteomics is challenged by both the presence of thousands of low-abundance proteins as well as the high dynamic range of protein concentrations [59]. Up to 99.9% of plasma proteins consist of the 21 most abundant ones, while the remaining 0.1% includes about 50,000 low-abundance proteins, often referred to as the “deep proteome”, where potential biomarkers may be found [60]. As a result, differential proteomics of unfractionated plasma provides limited insight. To increase analysis sensitivity, various precipitation methods [61] and commercially available depletion/enrichment strategies have been developed. These methods either remove the most abundant proteins or enrich plasma for low-abundant ones, mainly using antibodies, ion exchange, or affinity methods [62]. However, all these protocols may cause nonspecific co-depletion of less abundant proteins to some extent.

Several serological markers have been identified as correlating with coronary artery disease (CAD), covering a wide spectrum of pathophysiological processes such as cardiomyocyte injury, inflammation, oxidative stress, hemostasis, lipid metabolism, cell/cell interactions, and renal function. These biomarkers, relevant to ACS, are summarized in Table 1.

High-throughput unbiased quantitative plasma proteomics has also been applied to the identification of subclinical atherosclerosis biomarkers. In particular, Núñez et al. [76] performed an impressive study with a discovery phase on 444 subjects, well characterized by imaging of major arterial roots (carotids, infrarenal abdominal aorta, and iliofemoral arteries), cardiac computed tomography, clinical and blood parameters, and an external validation on 350 subjects. The identified panel of three proteins (Ig alpha-2 chain C region, apolipoprotein(a), and haptoglobin), assessed by standard assays on a cohort of 2999 subjects, was proven to be significantly associated with subclinical atherosclerosis.

In recent years, proteomics of secretory vesicles, intraluminal thrombus, urine, and purified lipoprotein fractions (i.e., lipoproteomics) has gained increasing attention as an alternative to whole plasma analysis in the search for new cardiovascular biomarkers.

### 2.1. Proteomics of Secretory Vesicles

Cells release various types of secretory vesicles, including microparticles, exosomes, and apoptotic bodies, found in all body fluids. These vesicles play relevant roles in intercellular communication by transferring proteins, nucleic acids, lipids, and metabolites to target cells via specific membrane receptors, and consequently, they differ in biogenesis, size, cargo, and biological functions. Understanding their roles in various pathophysiological processes can provide valuable insights into disease mechanisms and be key to developing novel therapeutic strategies for conditions such as cancer and cardiovascular disorders. Furthermore, the circulating levels of these vesicles could serve as biomarkers to assess disease severity and monitor treatment responses.

Secretory vesicles are of particular interest in the context of atherosclerosis, as they are released by multiple cell types involved in plaque progression, such as endothelial cells, smooth muscle cells, lymphocytes, monocytes, erythrocytes, and platelets, in response to pro-inflammatory signals and environmental stimuli like shear stress, hypoxia, and oxidative stress. These vesicles play a role in several pro-atherogenic events, including endothelial dysfunction, thrombosis, regulation of vascular tone, and the promotion of interactions between monocytes and endothelium, leukocytes and leukocytes, and leukocytes and endothelium. This has been consistently reviewed in the literature [47,77,78,79].

### 2.2. Proteomics of Intraluminal Thrombus

The formation of an intraluminal thrombus, triggered by a ruptured or eroded plaque, is considered the primary event leading to impaired blood flow in the coronary arteries during AMI. However, the exact mechanisms behind the sudden occlusion of coronary arteries in AMI remain unclear.

In a differential proteomics analysis, Distelmaier K et al. compared systemic plasma with plasma collected from the site of thrombus formation in AMI patients. Using two-dimensional gel electrophoresis followed by LC–MS/MS, they discovered, for the first time, an accumulation of the complement activator C-reactive protein, as well as the downstream complement effectors C3a and C5a, at the site of coronary thrombus formation during AMI. This localized event was shown to amplify the acute vascular occlusion process by enhancing neutrophil recruitment into the thrombus [80].

Alonso-Orgaz et al. conducted the first proteomic analysis of thrombus samples obtained from patients with STEMI through percutaneous intracoronary thrombectomy during primary angioplasty. By employing both gel-based and gel-free proteomic techniques, they identified 708 proteins in the thrombus, including a panel of five proteins—fermitin homolog 3, thrombospondin-1, myosin-9, beta parvin, and ras-related protein Rap-1b—that were positively correlated with the platelet marker CD41. These findings suggest the potential activation of a focal adhesion pathway in the platelets present within the thrombus. Additionally, the protein death-inducer obliterator 1 was found to be elevated in the plasma of STEMI patients, highlighting its potential as a systemic biomarker for thrombosis [81].

### 2.3. Urine Proteomics

Urine is an easily accessible and stable body fluid that remains resistant to proteolytic degradation even after extended storage, making it a valuable source of information. The protein and peptide composition of urine results from glomerular filtration and proximal tubular absorption of circulating proteins (about 30%) and from the urogenital tract (approximately 70%) [82]. Since urinary proteins can originate from all organs, reflecting both local and systemic pathophysiological conditions, urine has become one of the most promising biofluids in clinical proteomics, with applications across a wide range of medical fields. However, the urinary proteome is highly influenced by inter-individual and intra-individual variability, the latter being affected by factors such as physical activity, diet, medications, and caffeine consumption [83].

Although research on the urinary proteome in relation to CAD is still in its early stages, significant progress has been made in recent years [84]. Using capillary electrophoresis coupled with micro time-of-flight mass spectrometry, Delles et al. reported a panel of 238 CAD-specific polypeptides with high diagnostic potential [85,86,87]. This panel reverted to a physiological state following two years of antihypertensive treatment, suggesting its potential utility in patient monitoring. In a subsequent well-controlled prospective study, the same team identified a subset of markers predictive of CAD outcomes, including extracellular matrix components and proteins involved in hemostasis, erythropoiesis, inflammation, immune response, and signaling [88].

### 2.4. Lipoproteomics

Lipoproteomics is a specialized field of proteomics that focuses on the comprehensive analysis of apolipoproteins and lipoprotein-associated proteins, examining their structural and functional characteristics. It also aims to identify novel biomarkers that can be used for risk assessment, diagnosis, and treatment of various pathological conditions, particularly CVD.

Lipoproteins are supramolecular complexes that transport insoluble lipids from their sites of synthesis to tissues involved in their metabolism or storage. These complexes typically feature a hydrophobic core composed mainly of triacylglycerols and cholesteryl esters. Surrounding this core, there is a shell composed of amphipathic compounds, including phospholipids, unesterified cholesterol, and proteins, which are referred to as apolipoproteins (apo) and lipoprotein-associated proteins [89]. The various lipoprotein species found in circulation differ in their chemical composition, physical properties, and metabolic functions, and have been historically classified based on their densities: chylomicrons (<0.95 g/mL), very low-density lipoproteins (VLDL, <1.006 g/mL), low-density lipoproteins (LDL, 1.019–1.063 g/mL), intermediate-density lipoproteins (IDL, 1.006–1.030 g/mL), and high-density lipoproteins (HDL, 1.063–1.21 g/mL) [90]. Lipoprotein(a) or Lp(a) is another distinct class of lipoproteins, which consists of an LDL particle bound to a large, highly polymorphic glycoprotein called apo(a), connected to apo B100 by a single disulfide bond [91]. Smaller isoforms of apo(a), containing 11–22 Kringle IV repeats, have been strongly linked to high Lp(a) levels and an increased risk of coronary heart disease [92].

Lipoproteins, especially LDL and HDL, are crucial in the development of CVD. LDL is the primary transporter of cholesterol to peripheral tissues and a well-established major risk factor for atherosclerosis [22]. According to the “response-to-retention” hypothesis, LDL is retained in the subendothelial space of the arterial wall through interactions with ECM components, initiating the process of atherogenesis [93]. Conversely, HDL offers vascular protection by removing excess cholesterol from cells and facilitating its transport to the liver through reverse cholesterol transport [94]. Additionally, the pleiotropic HDL exhibits antioxidant, anti-inflammatory, and anti-thrombotic properties [95] and appears to play a role in innate immunity [96].

Recent studies have highlighted the critical role of lipoproteins’ protein and lipid components in influencing their functions in health and disease, particularly in CVD. Understanding the detailed composition and structure of the cargo within each lipoprotein class could provide valuable insights into their involvement in atherogenesis and the development of atherosclerotic plaques. In this context, the method used to isolate different lipoprotein particles is crucial, as it can significantly impact their protein content [97]. Ultracentrifugation in high-salt media represents a widely used method, established in the 1950s [98]. An issue of this approach is represented by the high ionic strength of the medium and the high centrifugal field forces that might cause either the dissociation of proteins or their exchange between different lipoprotein classes, potentially altering the pattern of associated exchangeable apolipoproteins. Additionally, some protocols have been set up in which salts were partially substituted by compounds, such as sucrose, iodixanol, or deuterium oxide. In this respect, Stahlman et al. suggested that deuterium oxide should be preferred over salts for isolating both LDL and HDL [99]. Alternatively, lipoproteins can be isolated through immunopurification methods that rely on specific antibodies for the dominant protein of each class. Although this procedure does not lead to the loss of weakly associated proteins, it may copurify non-specifically associated proteins, such as serum contaminants and/or other lipoprotein fractions carrying the same antibody target (e.g., apolipoprotein A-I is the main HDL apolipoprotein, but it is also present in both VLDL and LDL fractions). Other lipoprotein isolation methods that have been applied in lipoproteomic studies involve electrophoretic techniques, specifically free solution isotachophoresis [100], and chromatographic techniques, such as fast protein liquid chromatography [101], and size exclusion/affinity chromatography [102,103].

**Figure 5:**
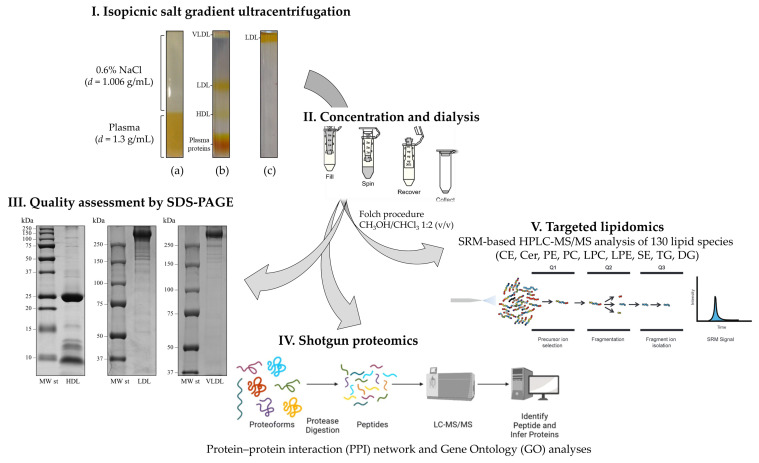
Example of methodological workflow for the molecular characterization of the different lipoprotein classes. (**I**) Isopycnic salt gradient ultracentrifugation. (**a**) Ultracentrifugation tube containing 0.9 mL of plasma sample combined with NaBr, overlaid with 2.1 mL of a 0.6% NaCl solution; (**b**) self-generated density gradient following ultracentrifugation at 541,000× *g* for 3 h at 4 °C in a TL-100 series ultracentrifuge equipped with a TLA-100 fixed-angle rotor (Beckman Coulter, Brea, CA, USA), showing the three main lipoprotein fractions; (**c**) LDL on the top of the tube following a further flotation step at d = 1.063 g/mL. (**II**) Concentration and dialysis using Amicon Ultra-0.5 mL centrifugal filter units (10 KDa MWCO, Merck-Millipore, Darmstadt, Germany). (**III**) Quality assessment using sodium dodecyl sulfate–polyacrylamide gel electrophoresis (SDS-PAGE). Representative mono-dimensional profiles of HDL, LDL, and VLDL fractions obtained by SDS-PAGE in either 12% T (for HDL, under reducing conditions) or 6% T (for both LDL and VLDL, under non-reducing conditions) resolving gels. (**IV**) Identification of the specific protein signatures of each lipoprotein fraction by tryptic digestion followed by LC-MS/MS and of the putative functions, biological processes, and interactions through protein/protein interaction (PPI) networks and gene ontology (GO) analyses [105]. (**V**) Identification of specific lipidomics patterns through selected reaction monitoring-based high-performance liquid chromatography-tandem mass spectrometry (SRM-based HPLC-MS/MS) analysis [106].

Furthermore, factors such as the choice of analytical method (instrumentation, quantification, and statistical approaches) and sample heterogeneity contribute to the discrepancies observed across studies, complicating the identification of widely accepted biomarkers (Figure 5) [104].

Notably, Davidson and colleagues have made significant contributions by curating a comprehensive list of proteins found in HDL and LDL, ranked by the frequency of their identification in mass spectrometry studies. This updated list is freely available to the scientific community through their website (www.DavidsonLab.com). The “HDL Proteome Watch Database” includes 285 proteins identified as “likely” HDL proteins, based on reports from at least three different laboratories out of a total of 1030 proteins identified across 51 studies. Gene ontology analyses of these proteins revealed their involvement in various biological processes, including lipid transport, hemostasis/protease inhibition, inflammation/acute phase response, immunity/anti-microbial functions, and cell/heparin binding [104]. Similarly, the database lists 60 proteins identified in four LDL proteomics studies up to 2015, with 22 of these proteins categorized as “likely” LDL proteins, because of independently identified by at least two studies. These include apolipoproteins A-I, A-II, A-IV, B, C-I, C-II, C-III, C-IV, D, E, F, J, L1, M, (a), serum amyloid A1, A2, A4, albumin, alpha-1-antitrypsin, cathelicidin antimicrobial peptide, dermcidin, and fibrinogen alpha chain.

Only a limited number of studies have focused on the VLDL proteome [99,107,108,109,110,111,112]. Among these, Dashty et al. identified 95 VLDL-associated and 51 LDL-associated proteins, of which 39 were common to both, and 56 and 12 were unique to VLDL and LDL, respectively. This set included all known apolipoproteins, lipid transport proteins, coagulation proteins, complement system proteins, and anti-microbial proteins [113].

Limited data are available on the Lp(a) proteome. Von Zychlinski et al. used two-dimensional liquid chromatography coupled with tandem mass spectrometry on highly purified Lp(a) particles that underwent tryptic digestion, identifying 35 proteins with high confidence. These proteins were involved in two key biological processes: lipid metabolism and response to wounding, the latter encompassing coagulation, complement activation, and inflammatory responses [114]. Bourgeois and colleagues conducted a discovery analysis on both Lp(a) and LDL, identifying 154 associated proteins. A differential analysis between Lp(a) and LDL proteomes revealed 15 proteins preferentially associated with Lp(a), which may be implicated in the acute inflammatory response, extracellular structure organization, and protease inhibition [115]. More recently, Mueller et al. developed a method for isolating Lp(a) from very small volume plasma samples (0.4 mL), enabling in-depth mass spectrometry analysis and providing a valuable tool for proteomics in case of limited sample availability [116].

Over the past two decades, numerous proteomic studies of purified HDL fractions have been conducted in relation to CAD, AMI, and ACS (Table 2).

While proteomics has advanced our understanding of the functions of different lipoprotein classes, particularly in relation to CVD, more information is needed to assess the residual risk of acute clinical events. The pleiotropic functions of lipoproteins, especially HDL, are influenced by both their protein and lipid components. Alterations in any of these elements can result in dysfunctional lipoproteins with pro-inflammatory and pro-atherogenic properties [129].

Recent years have seen a surge of interest in plasma lipidomics, driven by compelling evidence linking specific lipid species to the development of atherosclerosis [53,130,131,132,133,134,135,136], as well as to the onset of adverse clinical events [54,137,138,139,140,141]. However, only a limited number of studies have explored the relationship between biologically active lipids, their lipoprotein carriers, and CVD, as also recently reviewed by Ding and Rexrode [142].

Some studies have identified changes in the HDL phospho- and sphingolipidomes in connection with various pathological conditions strongly associated with CVD, such as type 1 and type 2 diabetes [143,144,145], obesity and metabolic syndrome [146,147], dyslipidemia [148,149], carotid atherosclerosis [106], and experimental atherosclerosis [150,151]. Furthermore, treatments such as statins [152] and dietary interventions with phytosterols and omega-3 fatty acids [153] have been shown to induce changes in the LDL lipidome, leading to a reduction in cardiovascular risk.

Lipidome profiles of both HDL and LDL have been explored in relation to CAD and ACS [154,155,156,157], revealing distinct differences when compared to healthy controls. Notably, the HDL phosphosphingolipidome exhibited unique signatures in individuals with hyperalphalipoproteinemia, a rare condition marked by elevated plasma HDL-cholesterol levels and an increased risk of early CAD [158,159].

To gain a more comprehensive understanding of lipoprotein metabolism in relation to CVD, Wang and colleagues recently developed a high-resolution method to analyze both the proteome and lipidome of lipoproteins and their interconnections [55]. Their findings demonstrated that the combined proteomic and lipidomic data provided better differentiation between ACS patients and healthy individuals than either proteomics or lipidomics alone.

## 3. Dissecting Atherosclerotic Plaque Through Proteomics

While tissue analysis can provide valuable insights, studying human atherosclerotic plaque specimens presents considerable challenges. Atherosclerotic plaques are complex tissues composed of various cell types within an environment marked by inflammatory, proteolytic [28,29], and oxidative processes [30,31]. These plaques reflect a systemic condition shaped by prolonged exposure to genetic and environmental risk factors. In addition to vascular smooth muscle cells (SMCs) and endothelial cells, plaques contain inflammatory cells, plasma proteins, newly synthesized ECM components, cell debris from apoptotic or necrotic events, and end-products of lipid and protein oxidation [18,24,160].

One potential solution to this issue is laser-captured microdissection (LCM), which allows for the isolation of specific cells or regions of the plaque under direct microscopic visualization. This technique has been minimally explored in proteomics but holds great promise. Another critical aspect of in situ analysis is selecting an appropriate control. To reduce tissue variability, it is ideal to use control samples from the same vascular region of the patient. Additionally, specimens obtained from surgical endarterectomy are preferred over post-mortem samples to avoid degradation of tissue before analysis. A key limitation, however, is the availability of human specimens. Given the heterogeneity of advanced lesions—such as variations in fibrous cap thickness, inflammatory and proteolytic components, calcifications, surface erosion, thrombosis, and intraplaque hemorrhage—a careful histochemical classification before biochemical analysis is essential [161,162].

In recent years, various proteomic technologies have been applied to diseased human tissues, providing deeper insights into the molecular mechanisms underlying advanced atherosclerotic plaque development. These studies have also enabled the identification of diagnostic features associated with plaque instability, which may serve as valuable therapeutic targets or biomarkers for patient monitoring. Research has primarily focused on both plaque extracts and secretomes, with the latter being derived by culturing different types of plaque segments. Additionally, animal models have been employed to further investigate the mechanisms involved in the early stages of lesion formation.

### 3.1. Proteomics of Coronary Plaque Extracts

Since the early 2000s, approximately twenty proteomics studies on human atherosclerotic plaques have been conducted, with a primary focus on either coronary (Table 3) or carotid atherosclerosis [163]. Both in-gel electrophoresis and shotgun proteomics have been employed to identify differentially expressed proteins. However, some limitations of these studies include the relatively small sample sizes and the challenges in selecting appropriate controls, which are often derived from unaffected vascular regions of the same patient or from different individuals. As a result, topological and inter-individual differences may be overlooked. Additionally, comprehensive validation using an independent cohort and complementary methodologies is often lacking.

The first large-scale protein profile of human atherosclerotic coronary arteries was provided by Bagnato et al., who identified 806 unique proteins with high confidence [165]. They utilized the direct tissue proteomic (DTP) approach, a technique developed by Hwang et al. for identifying candidate markers from formalin-fixed paraffin-embedded cancer specimens [169], to analyze paraffin and frozen tissue blocks from 35 coronary atherosclerotic lesions, which were classified histopathologically into early, intermediate, and advanced stages. The researchers laser-microdissected different plaque areas from both paraffin and frozen sections and subjected them to tryptic digestion followed by LC-MS/MS for area-specific proteomic analysis. Additionally, they homogenized the frozen sections, resolved the extracted proteins via SDS-PAGE, and analyzed them by LC-MS/MS. To quantify Stromal Cell-derived Factor 1 α and growth factors, which were not detected by the previous methods, they employed the AQUA (absolute quantification) methodology. This study demonstrated that laser capture microdissection (LCM) applied to plaque proteomics is an effective tool for comparing different plaque areas, such as the necrotic core and fibrous cap, providing valuable spatial insights.

LCM was also utilized by de la Cuesta et al. to isolate the intimal layers from human atherosclerotic coronary arteries, as well as from pre-atherosclerotic coronary and radial arteries, which were subsequently analyzed using 2D-DIGE coupled with MALDI-TOF/TOF MS for protein identification [166]. Their analysis revealed altered expression of 13 proteins involved in critical processes of plaque development, including vascular smooth muscle cell (VSMC) migration, ECM composition, coagulation, apoptosis, heat shock response, and intraplaque hemorrhage deposition.

In an effort to identify novel markers of plaque disruption, Lee et al. performed proteomic analysis on plaque debris obtained during coronary angioplasty using filterwire devices, identifying 423 proteins. The control group consisted of patients who underwent angiography without stenting [167]. They found that matrix metalloproteinase-9 (MMP-9) was significantly increased in lipid-rich plaques, as well as in the plasma of patients after plaque disruption by stenting. This novel method offers promising potential for discovering circulating biomarkers of plaque erosion, which is a key pathological mechanism behind major acute clinical events.

Although atherosclerosis often remains asymptomatic for many years, early lesions can manifest in children and young adults. Identifying arterial protein networks and their changes during the early stages of atherosclerosis holds great promise for diagnostics and therapy, with potential implications for preventing acute clinical events. In this context, Herrington et al. conducted a study to explore features of early atherosclerosis by examining 200 coronary and aortic autopsy samples from young adults. This approach led to the identification of 1925 proteins, as well as several networks and pathways associated with early atherosclerosis. Based on these findings, they selected a set of 13 plasma proteins, which were later shown to be effective in predicting the onset of CAD in an independent clinical cohort [33].

### 3.2. Proteomics of Plaque Secretomes

The plaque secretome may serve as another valuable source of biomarkers with potential clinical applications. It encompasses the full spectrum of proteins released or secreted into a serum-free medium by the plaque during culture. As such, the secretome is expected to reflect tissue-specific metabolic functions and changes in both healthy and diseased states. In comparison to tissue extracts and plasma samples, the secretome typically contains fewer protein species and exhibits a more limited range of protein concentrations.

Proteomics applied to the study of the cultured plaque secretome was initially pioneered by the teams of Vivanco and Egido, and this approach has since been successfully adopted in several studies [163]. The method developed by Duran et al. enabled the analysis of secretomes from different regions of the same carotid specimens obtained through endarterectomy (including normal segments, non-complicated plaques, and plaques complicated by thrombus). This approach helped mitigate intra- and inter-individual variability in control specimens by using 2D electrophoresis coupled with mass spectrometry [170]. Martin–Ventura and colleagues applied this technique to analyze protein secretion profiles from both femoral and carotid plaques, identifying Heat Shock Protein 27 as a potential circulating marker for atherosclerosis [171]. Notably, this method also proved valuable in assessing the effects of pharmacological treatments on plaque. In fact, the same team found that incubating plaque specimens with atorvastatin caused the expression of 66% of the proteins to revert to their baseline control levels [172].

De la Cuesta et al. conducted LC-MS/MS analysis on secretomes from human atherosclerotic coronary arteries, pre-atherosclerotic coronary arteries, and mammary arteries, identifying 64 proteins, 15 of which had not been previously reported in plasma. Due to the reduced dynamic range of protein concentrations in the secretome, they were able to identify low-abundance proteins such as laminin A/C, GELS, and vinculin, which are known to be involved in inflammation, cell adhesion, and arterial aging. These proteins were found to be significantly dysregulated in the coronary plaque secretome compared to the mammary artery secretome, suggesting potential therapeutic implications [173].

Secretome proteomics has also been applied to study coronary plaques in pigs fed a standard or high-cholesterol diet. This model, which closely mimics human pathology, offers valuable insights into the molecular mechanisms driving early atherogenesis, a stage that is challenging to study in human clinical settings. The early stages of plaque development are particularly difficult to examine in humans, but using a systems biology approach that integrates secretome data with histopathological features and circulating factors, Pelosi et al. demonstrated that systemic inflammation was locally associated with macrophage/phagocytosis-related protein expression in arteries, influencing atherosclerosis severity [51].

Rocchiccioli et al. mapped 224 proteins secreted by coronary plaques cultured from the same pig model used by Pelosi, finding a strong correlation between VSMC activation/migration and lesion progression [52]. Among the differentially expressed proteins associated with various stages of atherosclerosis (from type I to type V, according to Stary’s classification), chitinase 3-like protein 1 was identified, and its expression was validated through immunohistochemistry. This protein was proposed as a potential diagnostic marker for atherosclerosis.

## 4. Conclusions

The continuous discovery and understanding of new biomarkers for atherosclerosis hold the potential to significantly improve our ability to predict future cardiovascular risks, particularly when emerging biomarkers are integrated with traditional risk factors to assess overall risk profiles.

In recent years, proteomics has become a powerful tool in identifying novel cardiovascular biomarkers with diagnostic and prognostic significance. This approach offers an unparalleled opportunity to deepen our understanding of the various stages of atherogenesis and plaque development, bridging the gap between fundamental research and clinical applications. Plasma and plasma-derived fractions, as well as atherosclerotic tissues—especially plaque extracts and secretomes—are invaluable sources of information about both the early stages of atherogenesis and potential predictors of acute clinical events.

Despite its promise, advancing proteomics toward clinical practice faces several challenges. These include the intrinsic complexity of biological matrices, issues with sample availability, and the selection of appropriate controls, all of which can hinder results interpretation. Large-scale studies across different populations are needed to validate the clinical relevance of newly identified biomarkers, as well as the development of cost-effective standardized assays. While many databases exist that collect findings from genome-wide association studies and gene expression profiling, there is currently no proteomics-specific dataset dedicated to atherosclerosis. Therefore, future efforts must focus on thoroughly analyzing the wealth of data generated by proteomics studies to create comprehensive, shared datasets that include all identified biomarkers. These datasets should be integrated with data from other omics technologies, such as genomics, transcriptomics, and metabolomics. In this regard, artificial intelligence could play a crucial role in data analysis and interpretation, enabling fast, iterative processing of large volumes of information derived from these technologies. As technology advances and bioinformatics tools mature, proteomics is expected to play an increasingly pivotal role in precision cardiovascular medicine.

## Figures and Tables

**Table 1:** Plasma proteomics studies investigating CAD, acute myocardial infarction (AMI), and ACS. Reduced (↓) or increased (↑) levels of protein concentrations are indicated.

Plasma Samples	Proteomics Approach	Main Findings	References
11 AMI vs. 8 unstable angina vs. 9 healthy controls	2-DE coupled to MALDI-TOF MS	↓ alpha1-antitrypsin, ↑ Fibrinogen gamma chain, ↓ apolipoprotein A-I, ↑ ɣ-immunoglobulin heavy chains	[63]
53 pooled CAD vs. 53 pooled controls	albumin and immunoglobulin removal, low-molecular weight proteins enrichment, cation exchange, and reverse phase chromatography fractionation, LC-MS/MS	95 differentially expressed proteins, including inflammatory mediators, defense mechanism proteins, coagulation system proteins, and growth factors	[64]
48 non-ST elevation ACS vs. 10 CAD vs. 20 controls (6 months follow-up)	six most abundant proteins depletion, 2-DE and 2D-DIGE, MALDI-MS/MS	33 differentially expressed proteins belonging to the following functional groups: coagulation proteins, metabolism and/or lipid transport, inflammation and immune response, metabolite transport, cytoskeleton, miscellaneous proteins	[65]
800 pooled CAD vs. 800 pooled stroke vs. 1600 pooled controls (postmenopausal women)	six most abundant proteins depletion, protein labeling with C13 acrylamide, anion exchange, and reverse phase chromatography fractionation, LTQ-FT MS	beta-2 microglobulin and insulin-like growth factor binding protein 4 as risk markers for CHD and stroke, respectively; a number of candidate markers of disease risk and candidate mediators of hormone therapy effects on CHD and stroke	[66]
5 coronary bypass surgery patients (30-day follow-up)	2-DE coupled to LC-ESI-MS/MS	↑ α1-ACT and cathepsin G levels, which reflect a post-surgery pro-inflammatory and prothrombotic state due to neutrophil activation	[67]
22 ACS vs. 23 patients with normal coronary angiogram	2-DE coupled to nano LC-MS/MS	↑ serum amyloid A protein, ↑ alpha-1-antitrypsin, and fibrinogen A and B chains	[68]
409 ACS (3-year follow-up)	SELDI-TOF MS (SELDI protein chip technology)	m/z 4174.39 peak is associated with an increased incidence of 3-year events	[69]
30 pooled ACS vs. 30 pooled stable atherosclerotic disease vs. 30 pooled subclinical CVD vs. 30 pooled controls	albumin, IgG, IgA, haptoglobin, transferrin, and antitrypsin depletion; 4-plex iTRAQ followed by LC–MS/MS	increased expression of serum amyloid protein A (SAA), C-reactive protein (CRP), apolipoprotein(a), and vinculin, from controls to ACS	[70]
30 STEACS vs. 30 NSTEACS vs. 30 controls	14 most abundant plasma proteins depletion, 2D-DIGE, MALDI-TOF/TOF	differential expression of proteins involved in metabolism (~3.2%), lipid metabolism and transport (~29.3%), inflammation and immune response (~25.8%), blood homeostasis and coagulation (~16.2%), proteases and protease inhibitors (~9.7%), others (~6.4%) and unknown (~6.4%)	[71]
10 AMI vs. 5 controls	albumin, IgG, IgA, transferrin, haptoglobin, and antitrypsin depletion, iTRAQ followed by LC–MS/MS	a panel of 12 higher and 19 lower expressed proteins in AMI; the top 3 biological process terms were regulation of cell proliferation, response to wounding, and wound healing	[72]
20 Apo CIII < 10 mg/dL CAD vs. 32 Apo CIII > 10 mg/dL CAD	solid phase extraction followed by LC–MS/MS	↑ Apo C-II, ↑ Apo E, ↑ retinol-binding protein 4, and ↑ vitronectin, ↓ alpha-1 antitrypsin	[73]
20 ACS vs. 20 healthy controls	six most abundant proteins depletion, LC-ESI-MS/MS	↑ hemopexin, ↑ leucine-rich-2-glycoprotein, ↑ vitronectin, ↓ fibronectin	[74,75]
50 ACS vs. 50 healthy controls	albumin, antitrypsin, haptoglobin, IgA, IgG, and transferrin depletion, LC–MS/MS	↑ α-1-acid glycoprotein 1, complement C5, leucine-rich α-2-glycoprotein, vitronectin, ↓ GELS	[75]

**Table 2:** Proteomic studies performed on purified HDL fractions, investigating CAD, AMI, and ACS. Reduced (↓) or increased (↑) levels of protein concentrations are indicated.

Lipoprotein Fractions	Purification	Proteomics Approach	Main Findings	References
Group 1: HDL from 20 healthy volunteers; Group 2: HDL3 from 6 controls and 7 CAD patients; Group 3: HDL3 from 32 controls and 32 CAD; HDL from atherosclerotic tissue	Ultracentrifugation/immunoaffinity chromatography	LC-ESI-MS/MS analysis	48 proteins identified in HDL involved in lipid metabolism, complement regulation, proteinase inhibition, and acute-phase response. ↑ Apo C-IV, ↑ paraoxonase-1, ↑ Complement C3, ↑ apo A-IV, ↑ apo E (confirmed by WB on HDL3 fraction from 64 subjects) in HDL3 from CAD. More than 100 proteins identified in HDL from atherosclerotic plaque.	[96]
Group 1: HDL3 from 6 CAD, 6 CAD + Statin/Niacin, 6 healthy controls; Group 2: HDL3 from 18 CAD, 18 CAD + Statin/Niacin, 12 months therapy	Sequential density ultracentrifugation	LC-FTICR MS	After 12 months of therapy, ↓ apo E, ↑ apo F, and ↑ phospholipid transfer protein; combined therapy may revert CAD-associated changes in HDL3 protein composition.	[117]
HDL2 from 18 CAD vs. 20 controls	Sequential density ultracentrifugation	MALDI-TOF-TOF MS and pattern recognition analysis; LC-MALDI-TOF/TOF MS	HDL2 from CAD contains methionine sulfoxide residues in apolipoprotein A-I and elevated levels of apolipoprotein C-III; specific proteomic signatures of HDL2 accurately classify CAD and control subjects.	[118]
HDL from 39 new-onset AMI-patients vs. 60 healthy individuals	Salt gradient ultracentrifugation	2-DE coupled with MALDI-TOF MS analysis	Altered glycosylation pattern in AMI-patients within the first 6 h after the onset of the event.	[119]
HDL from 10 healthy controls vs. 10 CAD vs. 10 ACS	Sequential ultracentrifugation	1-DE coupled with LC-ESI-MS/MS analysis	67 HDL-associated proteins identified; ↓ apo A-IV, ↑ SAA, ↑ Complement C3 in ACS vs. both CAD and controls.	[120]
HDL from CAD, ACS, and healthy controls	Sequential ultracentrifugation or gel filtration chromatography	LC-ESI-MS/MS analysis	↓ clusterin, ↑ apolipoprotein C-III in both CAD and ACS; HDL-proteome remodeling plays an important role in the altered functional properties of HDL (stimulation of proapoptotic pathways).	[121]
HDL_2_ and HDL_3_ from 40 ACS vs. 40 controls	Two-step discontinuous density-gradient ultracentrifugation	2-DE and 2D-DIGE analysis coupled with MALDI-TOF/TOF MS	17 differentially expressed HDL-associated proteins identified, shifting to dysfunctional HDL subfractions.	[122]
HDL from 21 CAD pre- and post-percutaneous transluminal coronary angioplasty (PTCA)	Immunoaffinity chromatography (anti-apo A-I antibodies)	Quantitative ^16^O/^18^O analysis/iTRAQ technology coupled with LC–MS/MS/system biology analysis	225 identified proteins; high protein variability in HDL composition between individuals; post-PTCA increase in two protein clusters that included several apolipoproteins, fibrinogen-like protein 1, and other intracellular proteins, and a decrease in antithrombin-III, annexin A1, and several immunoglobulins.	[123]
HDL from 20 subjects at risk for CAD: 10 patients had CAD and 10 did not	Sequential ultracentrifugation	Shotgun proteomic, glycomic, and ganglioside analyses using LC-MS	Combined HDL proteomic and glycomic profiles distinguished at-risk subjects with atherosclerosis from those without; CAD patients had ↓ apo A-I, ↓ apo A-II, ↓ apo E, ↓ SAA2, ↓ SAA4 (*p* = 0.007), ↑ sialylated glycans.	[124]
HDL from 5 chronic heart failure (CHF) patients vs. 5 controls	Sequential ultracentrifugation	SCX/RP LC–MS/MS	494 proteins identified; 23 newly identified HDL-associated proteins; 223 bacterial peptides were found in both CHF and controls.	[125]
HDL from 126 subjects with clinical indication for a coronary computed tomography angiography	High-resolution size exclusion chromatography followed by phospholipid-associated proteins capture (calcium silicate hydrate)	LC-ESI-MS/MS	72 HDL-associated proteins detected in at least 75% of subjects; 13 proteins significantly associated with calcified plaque burden including cathelicidin antimicrobial peptide, GELS, kininogen-1, and paraoxonase-1 (inverse relationships), apo A-IV, vitamin D binding protein, alpha-2- macroglobulin, and apo C-II (positive relationships); 15 proteins significantly associated with non-calcified plaque burden including apo A-I, apo F, antithrombin III, and apo C-I (inverse relationships), serum amyloid A1, immunoglobulin heavy constant alpha 1, complement factor B, complement C2, complement C3, complement bC1s subcomponent (positive relationships); among the evaluated risk factors, body mass index has the greatest overall impact on the protein composition of HDL.	[126]
HDL from 943 participants without prevalent myocardial infarction referred for coronary angiography in the CASABLANCA study	^15^NHis_6_Apo A-I was added to human serum, incubated, diluted, and then purified using PhyTips (Phynexus, San Jose, CA, USA), packed with Ni-NTA HisBind Superflow stationary phase	Targeted proteomics (apolipoprotein A-I, C-1, C-2, C-3, and C-4): LC-ESI-MS/MS analysis	An HDL apolipoproteomic score is associated with CAD, independent of circulating apo A-I and apo B concentrations and other conventional cardiovascular risk factors. Among individuals with CAD, this score may be independently associated with cardiovascular death.	[127]
Apolipoprotein AI-associated lipoproteins from 231 healthy individuals and patients with CAD	Metal chelate affinity chromatography	Targeted proteomics (21 proteins): LC-MS/MS analysis	A multiplexed proteomic assay useful for the estimation of cholesterol efflux and CAD risk in the clinical laboratory.	[128]

**Table 3:** Proteomics studies on human coronary plaque extracts. The sample source, the methodology applied, and the most relevant findings are reported.

Samples	Proteomics Approach	Main Findings	References
10 coronary arteries from CAD vs. 7 coronary arteries from normal individuals	2-DE coupled with LC-MS/MS	Increased expression of the ferritin light chain in CAD (1.41 vs. 0.75; *p* = 0.01).	[164]
35 human coronary atherosclerotic specimens (frozen, embedded, LCM specimens)	LC-MS/MS	806 proteins identified; first large-scale proteomics map of human coronary atherosclerotic plaques.	[165]
Intimal proteome from the human atherosclerotic coronary artery (LCM) vs. preatherosclerotic coronary vs. radial arteries	2D DIGE coupled with MALDI TOF/TOF MS	13 proteins were differentially expressed (7 upregulated and 6 downregulated), and are implicated in the migrative capacity of vascular SMCs, ECM composition, coagulation, apoptosis, heat shock response, and intraplaque hemorrhage deposition.	[166]
23 lipid-rich plaques vs. 13 non-lipid-rich plaques	LC MS/MS	Library of 423 proteins that are present in coronary plaque debris; MMP-9 and another 5 plaque-enriched proteins (lipopolysaccharide binding protein, Annexin A5, eukaryotic translocation initiation factor, syntaxin 11, cytochrome B5 reductase 3) significantly enriched in plaque and in plasma after plaque disruption.	[167]
Human coronary arteries (n = 100) and aortas (n = 100)	LC MS/MS	Identified hundreds of proteins (n = 1925) and numerous networks and pathways that are associated with early atherosclerosis.	[33]
Coronary arteries at different stages of development, from 15 patients	2-DE followed by MALDI-TOF MS	The amounts of the following proteins were increased in stable atherosclerotic plaques at the stage of lipidosis and fibrosis: vimentin, tropomyosin β-chain, actin, keratin, tubulin β-chain, microfibril-associated glycoprotein 4, serum amyloid P-component, and annexin 5. In plaques at the stage of fibrosis and calcification, the amounts of mimecan and fibrinogen were increased. In the unstable atherosclerotic plaque of the necrotic–dystrophic type, the amounts of human serum albumin, mimecan, fibrinogen, serum amyloid P-component, and annexin were increased.	[168]

## Abbreviations

The following abbreviations are used in this manuscript:

CVD	cardiovascular disease
CAD	coronary artery disease
CHD	coronary heart disease
ACS	acute coronary syndrome
AMI	acute myocardial infarction
NSTEMI	non-ST-segment elevation myocardial infarction
STEMI	ST-segment elevation myocardial infarction
hs-CRP	high-sensitivity C-reactive protein
H-FABP	high-sensitivity C-reactive protein
CK-MB	creatine kinase-MB isoform
hs-cTn	high-sensitivity cardiac troponin
VSMC	vascular smooth muscle cell
VLDL	very low density lipoprotein
LDL	low-density lipoprotein
HDL	high-density lipoprotein
ECM	extracellular matrix
1-DE	one-dimensional electrophoresis
2-DE	two-dimensional electrophoresis
MS	mass spectrometry
LCM	laser capture microdissection
MALDI	matrix-assisted laser desorption ionization
ESI	electrospray ionization
SELDI	surface-enhanced laser desorption/ionization
TOF	time of flight
LC-MS/MS	liquid chromatography coupled to MS/MS
2D-DIGE	two-dimensional difference gel electrophoresis
LTQ-FT MS	hybrid mass spectrometer consisting of a linear ion trap mass spectrometer, LTQ XL, combined with a Fourier transform ion cyclotron resonance mass spectrometer
Ig	immunoglobulin
iTRAQ	isobaric tag for relative and absolute quantitation
Apo	apolipoprotein
α1-ACT	α1-antichymotrypsin
SAA	serum amyloid A protein
CRP	C-reactive protein
STEACS	ST-segment elevation acute coronary syndrome
NSTEACS	non-ST-segment elevation acute coronary syndrome
Lp(a)	lipoprotein(a)
LC-FTICR MS	liquid chromatography/Fourier transform ion cyclotron resonance mass spectrometry
PTCA	percutaneous transluminal coronary angioplasty
CHF	chronic heart failure
SCX/RP	strong cation exchange reverse
GELS	gelsolin
MMP	matrix metalloproteinase
SDS-PAGE	sodium dodecyl-sulphate polyacrylamide gel electrophoresis
PTMs	post-translational modification
pI	isoelectric point
MW	molecular weight
